# The predictive powers of plasma trefoil factor 3 or its related micro RNAs for patients with hepatocellular carcinoma

**DOI:** 10.1186/s12885-018-5017-y

**Published:** 2018-11-13

**Authors:** Chenghua Zhang, Ran Xia, Bo Zhang, Haibo Wang

**Affiliations:** 1 Department of Endoscopy, Jilin Cancer Hospital, Changchun, 130012 China; 2 Department of Geriatrics 1, Affiliated Hospital of Changchuan University of Traditional Chinese Medicine, Changchun, 130012 China; 3 Department of Hepatopancreaticobiliary Surgery, Second Affiliated Hospital of Dalian Medical University, 467 Zhongshan Road, Dalian, 116044 China

**Keywords:** Trefoil factor 3, MiR-7-5p, MiR-203a-3p, Biomarker, Hepatocellular carcinoma

## Abstract

**Background:**

Earlier diagnosis is beneficial for the prognosis of hepatocellular carcinoma (HCC). Alpha fetoprotein (AFP) is the most widely used biomarker for HCC, but its sensitivity and specificity are only 60 and 90%, respectively. Therefore, it is of great clinical significance to identify early prognostic biomarkers for HCC, especially a blood-based biomarker as it offers several advantages over tissue-based biomarkers. Trefoil factor 3 (TFF3), a novel secretory protein, was over-expressed in HCC tissues, indicating it might be a blood-based biomarker for HCC. In addition, circulating microRNAs have been investigated as biomarkers for HCC, indicating that miR-7-5p and miR-203a-3p, which are reported or predicted to target TFF3, also hold promise as blood-based biomarkers for HCC.

**Methods:**

We enrolled 43 patients who were firstly diagnosed HCC and matched 47 control subjects without HCC. The levels of TFF3, miR-7-5p and miR-203a-3p were tested in the plasma of HCC patients. Moreover, we assayed the correlation of TFF3 with its related micro RNAs, miR-7-5p and miR-203a-3p, and evaluated their predictive powers for HCC.

**Results:**

Decrease of TFF3 was associated with increase of miR-203a-3p in the plasma of HCC patients and they displayed potent predictive powers for HCC diagnosis. However, there was no significant change of plasma miR-7-5p between HCC and control group.

**Conclusion:**

Decrease of TFF3 correlated with increase of miR-203a-3p in the plasma of HCC patients and they could be additional biomarkers to improve sensitivity and specificity in the diagnosis of HCC.

## Background

Hepatocellular carcinoma (HCC), the main primary liver cancers, is the third lead causes of cancer death [1]. Earlier diagnosis is benefit for the prognosis of HCC. Alpha fetoprotein (AFP) is the most widely used biomarker for HCC, but its sensitivity and specificity are only 60 and 90%, respectively [2]. Therefore, there is an urgent need to identify early prognostic biomarkers for HCC. In addition, blood-based biomarker offers several advantages over tissue-based biomarker as it is simpler and less invasive [3].

Trefoil factor 3 (TFF3) is a novel secretory protein and plays an important role in tumor genesis and immunity [4]. Recently, over-expression of TFF3 was found in spontaneous and carcinogen-induced HCC of mice model or tissues of HCC patients and associated with higher tumor grade [5, 6]. Consistently, microarray analysis of HCC tissues from hepatitis B virus X (HBx) transgenic mice showed that the expression level of TFF3 gene was higher than AFP, suggesting that TFF3 might be as a blood-based biomarker for HCC [7]. In addition, it had been demonstrated that some circulating cancer biomarkers such as micro RNAs were from immune cells or other blood cells but not cancer tissues [8, 9]. And, their levels in blood and cancer tissues were not always consistent [8, 10]. We found that TFF3 gene was significantly down-regulated in peripheral blood samples of HCC patients by analyzing microarray data of GDS4882 including 10 patients with HCC and 10 normal subjects, which were obtained from the Gene Expression Omnibus database. Therefore, the predictive potency of circulating TFF3 for HCC needs further study. In addition, micro RNA could be as circulating biomarker as they were altered and stable in blood. Therefore, micro RNAs that target TFF3 might be also used as the biomarkers for HCC.

In this study, we investigated the plasma levels of TFF3 and miR-7-5p and miR-203-3p, which were reported or predicted to target TFF3, respectively [7, 8]. In addition, we analyzed the correlations of TFF3 with miR-7-5p or miR-203-3p, and their predictive powers for HCC by performing ROC (Receiver Operating Characteristic) curves and Binary logistical regression analysis.

## Methods

### Study population

In this study, we enrolled 43 HCC patients who were firstly diagnosed by computed tomography (CT) and/or magnetic resonance imaging (MRI) observations as well AFP assay and matched 47 control subjects without cancers at Second Affiliated Hospital of Dalian Medical University, Dalian China, From March 2015 to March 2016. All HCC patients were at the early stage and their blood samples (5 ml per patient) were collected before those received any treatments via a direct venous puncture and placed into tubes containing sodium citrate. To get plasma without cell pollution, all samples were first centrifuged at 3000×g for 5 min and then the layer was carefully transferred into other tubes which were centrifuged at 5000×g for 10 min. All plasma samples were stored at − 80 ∘C until use. Written consent was obtained from all subjects, and the study protocol was approved by the ethics committee of the Second Affiliated Hospital of Dalian Medical University (20180504007).

### GEO analysis of TFF3 gene expression in peripheral blood samples of HCC and prediction of miRNA targeting TFF3

Microarray data of GDS4882 including 10 patients with HCC and 10 normal subjects were obtained from the Gene Expression Omnibus database. Expression of TFF3 gene in peripheral blood samples of HCC patients was analyzed. MiRNAs targeting TFF3 was predicted using the publicly available TargetScan (http://www.targetscan.org).

### Assay for plasma TFF3, miR-7-5p and miR-203a-3p

We used the enzyme-linked immunosorbent assay (ELISA) kit from Elabscience to assay the level of TFF3 in the plasma of HCC patients, following the manufacturer’s instructions. Absorbance was measured at 450 nm (primary wave length).

Total RNAs were isolated from the plasma samples which were firstly added with 50 pmol/L *Caenorhabditis elegans* miR-39 (cel-miR-39), an external reference, following the instruction of miRcute miRNA Isolation kit (TRANS GEN, Beijing, China). The cDNAs were then generated by poly-(A) tailing and reverse transcription using the miScript reverse transcription kit (TRANS GEN, Beijing, China). QPCR was conducted with the ViiA 7 Software v1.1 (Applied Biosystems) to quantify miRNAs as follows: 95 °C for 5 min, followed by 40 cycles of 95 °C for 15 s and 60 °C for 30 s. Micro RNA assay primers used were miR-7-5p forward: 5′-TGGAAGACTAGTGATTTTGTTGTT-3′, miR-203a-3p 5′-GTGAAATGTTTAGGACCACTAG-3′ and cel-miR-39 forward: 5′-TCACCGGGUGUAAATCAGCTTG-3′. Negative controls using nuclease-free water were included with every real-time PCR operation and cycle threshold (CT) values ≤6 or > 35 were removed from analysis. All samples for miRs were run in one assay and all reactions were run in triplicate. Analysis of relative gene expression levels was performed using the formula 2-ΔCT with ΔCT = CT (target gene)-CT (control) [11].

### Statistical analyses

Statistical analyses were performed using SPSS 19.0. Data are presented as the mean ± SD and median for the general characteristics of the subjects. Differences among the different groups were assessed using the one-way ANOVA comparison method. Values with a *p* <  0.05 were considered to indicate statistical significance. The correlation of plasma TFF3 with its related micro RNAs were calculated using the Spearman correlation test. The predictive powers of plasma TFF3 with its related micro RNAs for HCC were assayed by receiver operating characteristic (ROC) and binary logistical regression analysis [11].

## Results

### Baseline characteristics

Fourty-three patients with HCC and matched 47 control subjects without HCC were enrolled. Their clinical characteristics and biochemical parameters are listed in Table 1. Age; sex; hypertension; smoking history; drinking history; Hepatitis B or Hepatitis C infection; were not different between HCC and control groups. The plasma level of AFP in HCC patients was significantly increased compared to control group. However, the level of AFP in 39.95% of HCC patients was less than its cut-off value (20 ng/ml) and the level of AFP in 8.51% of control subjects was higher than that.

**Table. 1:** Clinical characteristics and biochemical parameters of the patients

Variable	Control	HCC	*P* value
	*n* = 47	*n* = 43	
Age (y)	55.96 ± 11.15	55.40 ± 10.51	0.807
Male *n* (%)	38 (80.85)	29 (67.44)	0.148
Somking *n* (%)	13 (27.66)	12 (27.90)	0.979
Drinking *n* (%)	11 (23.40)	5 (11.63)	0.148
Hypertension, *n* (%)	7 (14.89)	6 (13.95)	0.901
Hepatitis B *n* (%)	10 (21.28)	13 (30.23)	0.336
Hepatitis C *n* (%)	2 (10.64)	3 (6.98)	0.578
AFP (ng/ml)	6.70 ± 13.10	274.14 ± 434.30	< 0.01

### Analysis of TFF3 gene expression in peripheral blood samples of HCC patients and screening of micro RNAs targeting TFF3

**Fig. 1:**
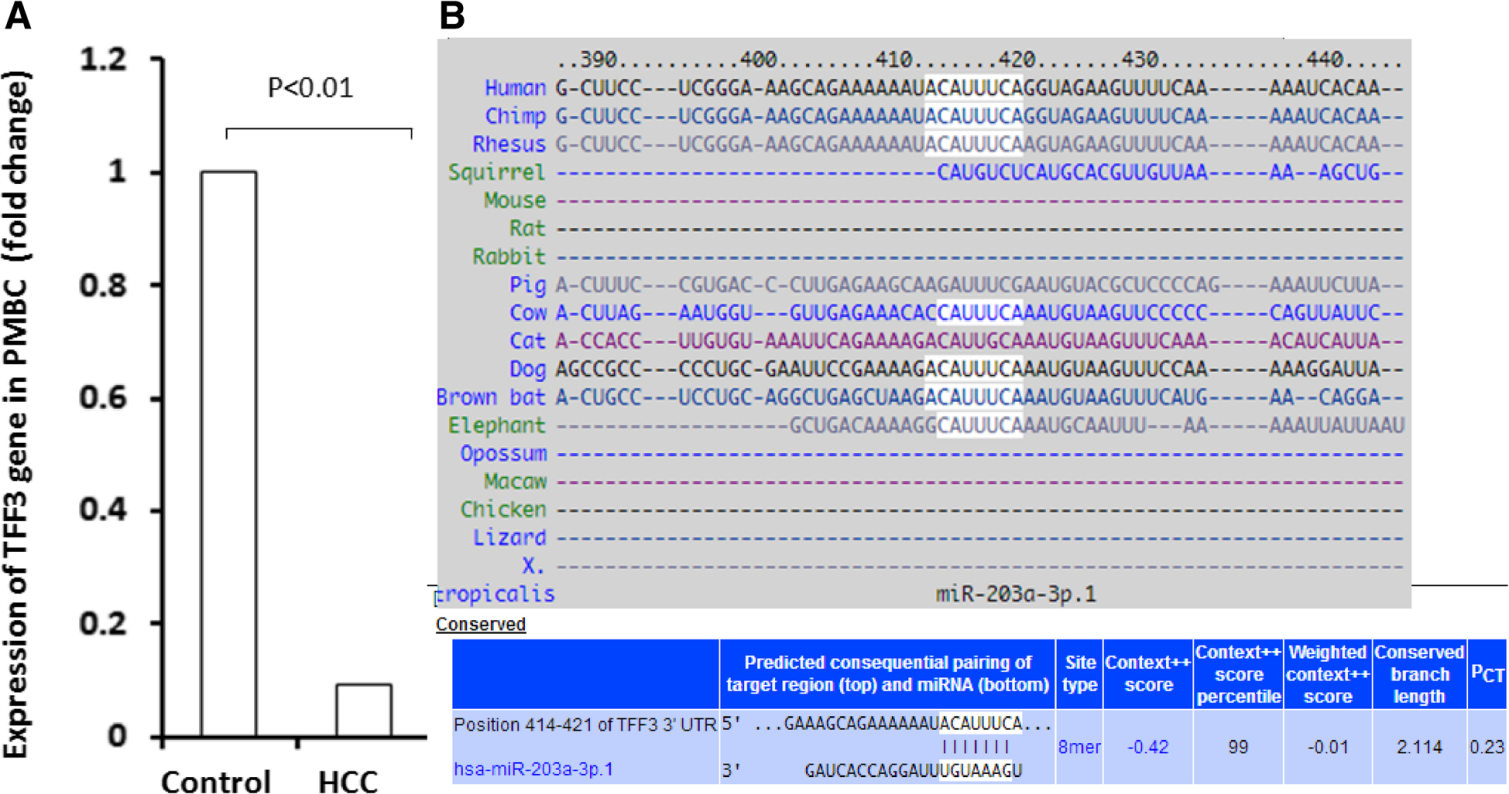
Analysis of TFF3 gene expression in peripheral blood samples of HCC patients and Screening of micro RNAs targeting TFF3. **a** The expression of TFF3 gene in peripheral blood samples of HCC patients by GEO data analysis, **b** prediction of micro RNA that target at TFF3 by screening TargetScan database

The expression of TFF3 gene was higher than AFP in microarray analysis of HCC tissues from HBx transgenic mice and protein level of TFF3 was higher in tissues of HCC patients [7]. In addition, TFF3 holds promising as a blood-based biomarker as it is a secretory protein. However, it was also reported that some circulating cancer biomarkers such as micro RNAs were not only from cancer tissues but also from the immune or other blood cells [8, 9]. And, their levels in blood and cancer tissues were not always consistent [10]. In this study, we also analyzed the expression of TFF3 gene in peripheral blood samples of HCC patients, the microarray data (GDS4882) was obtained from Gene Expression Omnibus database. The results showed that expression level of TFF3 gene in peripheral blood samples of HCC patients was 0.09 fold compared with that in control subjects (*p* <  0.01) (Fig. 1a).

MiR-7-5p has been proven to directly bind the 3’UTR of TFF3 mRNA. In addition, we screened TargetScan database, and predicted that hsa-miR-203a-3p was the only microRNA that could bind to converse site located with the 3’UTR of TFF3 mRNA (nt 414–421) with a high target score (Fig. 1b).

### The plasma levels of TFF3 and miR-7-5p, miR-203-3p and their correlations in HCC patients

**Fig. 2:**
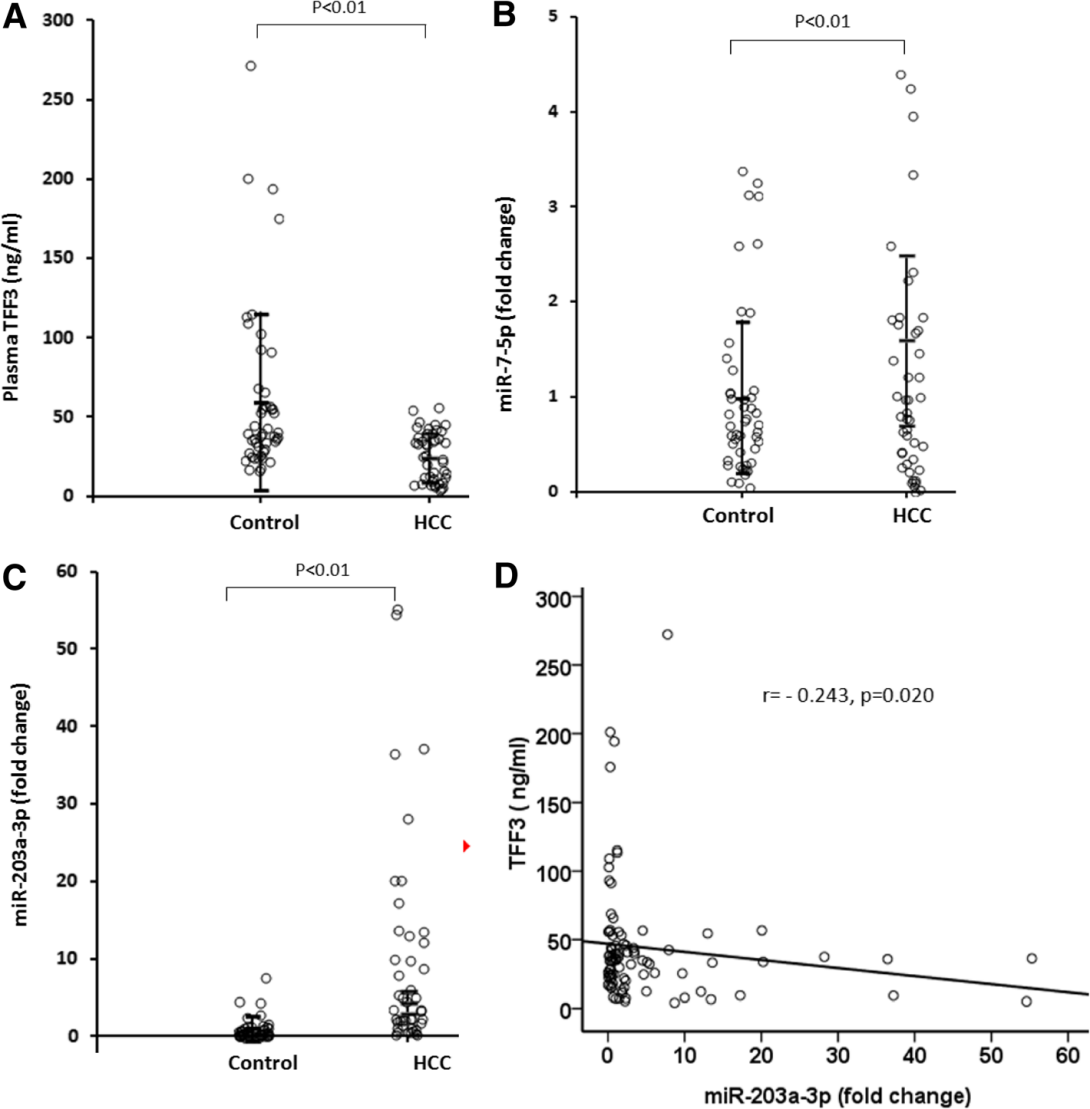
The plasma levels of TFF3 and miR-7-5p, and miR-203a-3p and the correlation of TFF3 and miR-203a-3p. **a** The plasma level of TFF3 in HCC patients or control subjects, **b** T The plasma level of miR-7-5p in HCC patients or control subjects, **c** The plasma level of miR-203a-3p in HCC patients or control subjects, **d** The correlation of plasmaTFF3 and miR-203a-3p

To explore the biological relevance of TFF3 and its related micro RNAs in HCC, we tested plasma levels of TFF3 and miR-7-5p, miR-203a-3p in HCC patients. The results showed that the plasma level of TFF3 was markedly decreased in HCC patients compared with control subjects (25.83 ± 15.53 vs 60.91 ± 53.93 ng/ml, *p* < 0.01) (Fig. 2a). In contrast, the plasma miR-203a-3p was significantly increased in patients with HCC compared with control subjects (9.57 ± 13.52 vs 1.00 ± 1.43 fold, *p* < 0.01) (Fig. 2c). While, there was no significant difference in plasma miR-7-5p between HCC and control group (1.18 ± 1.13 vs 1.00 ± 0.90 fold, *p* < 0.01) (Fig. 2b).

In addition, we analyzed correlations of TFF3 with miR-203a-3p and found that TFF3 negatively correlated with miR-203a-3p in the plasma of HCC patients (*r* = − 0.243, *p* = 0.020) (Fig. 2d).

### Comparison of the predictive powers of TFF3 and miR-203a-3p for HCC

**Fig. 3:**
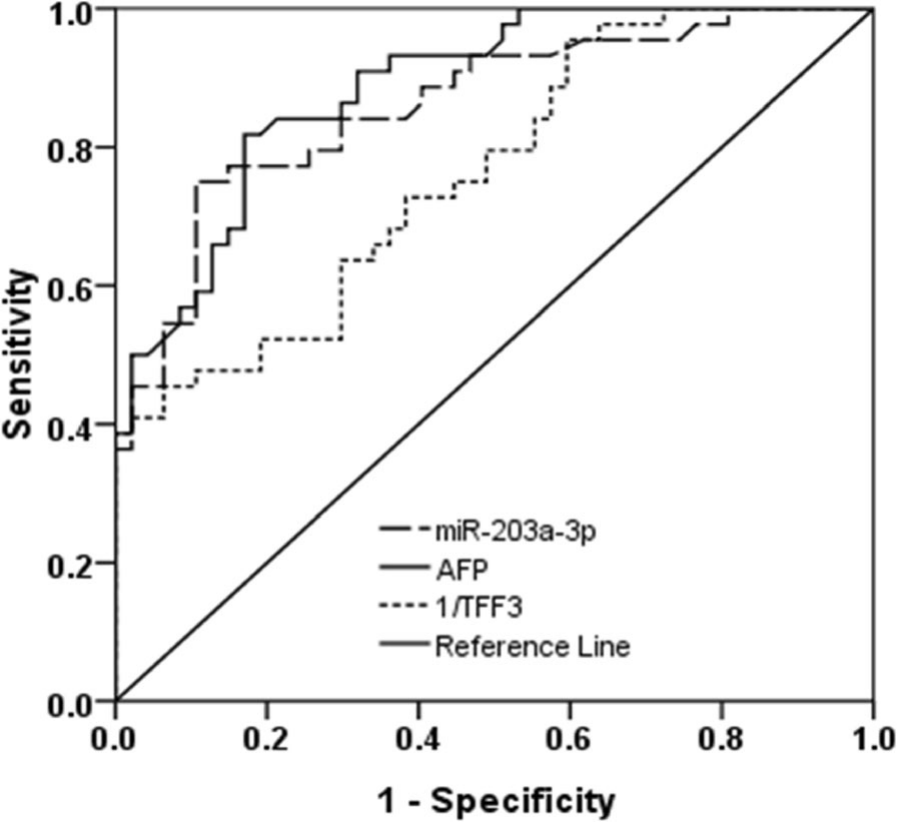
The ROC analysis of the predictive power of TFF3 and miR-7-5p, and miR-203a-3p for HCC status

We evaluated the predictive powers of plasma TFF3 and miR-203a-3p for HCC by receiver operating characteristic (ROC) and binary logistical regression analysis. The predictive power of AFP for HCC was also tested in this study, and the AUC for AFP was 0.886 (95% CI: 0.821–0.951; *p* = 0.00). The miR-203a displayed similar predictive potency with AFP, the AUC for miR-203a-3p was 0.860 (95% CI: 0.784–0.936; *p* = 0.00) and the optimal cut-off value was 1.44 fold with sensitivity and specificity of 77.3 and 75.1%, respectively (Fig. 3). As plasma TFF3 was down-regulated in HCC patients, we used 1/TFF3 to distinguish the HCC patients and the results showed that the AUC for 1/ TFF3 was 0.763 (95% CI: 0.667–0.859; *p* = 0.00) and the optimal cut-off value was 35.13 ng/ml with sensitivity and specificity of 70.2 and 63.6%, respectively (Fig. 3). Moreover, we also corroborated the discriminatory value of TFF3, miR-7-5p, miR-203a-3p, and AFP by binary logistical regression analysis (Table 2). The results showed that plasma TFF3, miR-203a-3p, and AFP were significantly correlated with HCC status (*p* = 0.028, *p* = 0.012, *p* = 0.037, respectively).

**Table 2:** Binary logistical regression analysis of TFF3, miR-7-5p, miR-203a-3p, AFP, in patients with HCC

Variable	*p*-value	OR	95% CI	
			Lower	Upper
TFF3	0.028	0.944	0.896	0.994
miR-7-5p	0.829	1.098	0.468	2.578
miR-203a-3p	0.012	1.805	1.14	2.857
AFP	0.037	1.042	1.002	1.084

## Discussion

TFF3 plays an important role in promoting proliferation and migration in many tumor cells such as mammary, prostate carcinoma and HCC cells [12–14]. Over-expression of TFF3 was found in HCC tissues and positively correlated with tumor size and stage [14–16]. TFF3 could promote angiogenesis in mammary carcinoma or in human umbilical vein endothelial cells via up-regulation of vascular endothelial growth factor (VEGF) [17, 18]. In addition, over-expression of TFF3 facilitated the doxorubicin resistance in gastric cancer and HCC cells, which limited the efficacy of chemotherapeutic treatment of HCC [19, 20]. Moreover, microarray analysis of HCC tissues from HBx transgenic mice showed that expression levels of TFF3 gene was higher than AFP, which was widely used as biomarker for HCC, indicating that TFF3 could be as biomarker for HCC [7]. Moreover, TFF3 holds promising as blood-based biomarker as it is secretory protein. Interestingly, we found the plasma level of TFF3 was reduced in HCC patients. Consistently, expression of TFF3 gene was down-regulated in peripheral blood samples of HCC patients by analyzing microarray data of GDS4882, which were obtained from Gene Expression Omnibus database, indicating that the plasma TFF3 might be secreted from immune cells or other blood cells.

Similarly, miRNAs with a relatively low expression in cancer tissue are abundantly expressed in plasma or serum of gastric cancer patients [8]. It has been demonstrated that only a small subset of circulating miRNAs are secreted from tumor and most of them are produced by immune and other blood cells [8–10]. In this study, we also investigated circulating miR-203a-3p, which was predicted to target TFF3 by bioinformatics analysis and found that miR-203a-3p was up-regulated and negatively correlated with TFF3 in the plasma of HCC patients. However, miR-203 was down-regulated in HCC tissue [19]. Low level of miR-203a was also found in many other types of tumor such as breast cancer and colorectal cancer, which was also positively correlated with proliferation, migration, invasion and angiogenesis [21–24]. In addition, miR-122 was down-regulated in HCC tissues and cancer cell lines [25] but up-regulated in the serum of HCC patients [7]. Previous observations also showed that only a few differentially expressed miRNAs were common to tumors and serum [26]. Therefore, the plasma miR-203 might be from the immune cells or other blood cells but not HCC tissues.

The abilities of TFF3 and miR-203 in regulating immune response are also found, which might participate in the development of HCC. It has been demonstrated that TFF3 could activate nuclear factor kappa B (NF-κB) in intestinal epithelial cells to produce pro-inflammatory cytokines [27]. Furthermore, TFF3 also acts as a negative regulator of T-cell immunity by induction of the expression of decay accelerating factor [28]. Therefore, low level of TFF3 in plasma or peripheral blood might exert an immunosuppressive effect and favor the development of HCC. MiR-203 could inhibit the inflammatory response induced by lipopolysaccharide in macrophage [29]. In addition, miR-203 was also found to inhibit the expression of TLR4 and production of tumor necrosis factor-α and interleukin-12 in dendritic cells [30]. Moreover, miR-203 could suppress the production of inflammatory cytokines and thereby prevented mounting of a full immune response [31]. Those data indicated that high level of plasma miR-203 might also inhibit immune response against HCC. The immune-regulatory roles of TFF3 or miR-203 need further study.

In addition, miR-7-5p was reported to target TFF3 in inflammatory bowel disease [32]. MiR-7 was down-regulated in HCC tissues and the low expression of miR-7 positively correlated with tumor size [33]. It was found that miR-7 inhibited proliferation of HCC via targeting Kruppel-like factor 4 [34]. However, our data showed that there was no significant difference of plasma miR-7-5p between HCC and control group. We further evaluated the predictive powers of plasma TFF3 and miR-203a-3p for HCC and they showed a highly significant diagnostic value for HCC. Notably, TFF3 and miR-203a-3p were altered in many other tumors, indicating that they might be as additional biomarkers for HCC.

Notably, there are some limitations in this study. For example, the plasma levels of TFF3 and miR-203a-3p and their predictive potencies were tested for HCC, but they might be also changed in many other tumors. Therefore, they might only be used as additional biomarkers for HCC. In addition, the sample size was small and need larger studies from multiple centers.

## Conclusion

Decrease of TFF3 correlated with increase of miR-203a-3p in the plasma HCC patients and they could be as additional biomarkers to improve sensitivity and specificity in the diagnosis of HCC.

## Abbreviations

AFP: Alpha-fetoprotein
HBx: Hepatitis B virus X
HCC: Hepatocellular carcinoma
miRNA: Micro RNA
ROC: Receiver operating characteristic
TFF3: Trefoil factor 3

## References

[1] Bruix J, Sherman M (2011). Management of hepatocellular carcinoma: an update. Hepatology.

[2] Ji J, Wang H, Li Y (2016). Diagnostic evaluation of des-gamma-carboxy prothrombin versus α-fetoprotein for hepatitis B virus-related hepatocellular carcinoma in China: a large-scale, multicentre study. PLoS One.

[3] Ganepola GAP, Nizin J, Rutledge JR (2014). Use of blood-based biomarkers for early diagnosis and surveillance of colorectal cancer. World J Gastrointest Oncol.

[4] Baus-Loncar M, Giraud AS (2005). Trefoil factors. Cell Mol Life Sci CMLS.

[5] Chen X, Yamamoto M, Fujii K (2015). Differential reactivation of fetal/neonatal genes in mouse liver tumors induced in cirrhotic and noncirrhotic conditions. Cancer Sci.

[6] Franke TF (2008). PI3K/Akt: getting it right matters. Oncogene.

[7] Sun Q, Zhang Y, Liu F, Zhao X, Yang X (2007). Identification of candidate biomarkers for hepatocellular carcinoma through pre-cancerous expression analysis in an HBx transgenic mouse. Cancer Biol Ther.

[8] Sierzega M, Kaczor M, Kolodziejczyk P (2017). Evaluation of serum microRNA biomarkers for gastric cancer based on blood and tissue pools profiling: the importance of miR-21 and miR-331. Br J Cancer.

[9] Wulfken LM, Moritz R, Ohlmann C (2011). MicroRNAs in renal cell carcinoma: diagnostic implications of serum miR-1233 levels. PLoS One.

[10] Pritchard CC, Kroh E, Wood B (2011). Blood cell origin of circulating microRNAs: a cautionary note for cancer biomarker studies. Cancer Prev Res.

[11] Fei Yu, Hou Jianhua, Xuan Wei, Zhang Chenghua, Meng Xiuping (2018). The relationship of plasma miR-503 and coronary collateral circulation in patients with coronary artery disease. Life Sciences.

[12] Kannan N, Kang J, Kong X (2010). Trefoil factor 3 is oncogenic and mediates anti-estrogen resistance in human mammary carcinoma. Neoplasia.

[13] Garraway IP, Seligson D, Said J, Horvath S, Reiter RE (2004). Trefoil factor 3 is overexpressed in human prostate cancer. Prostate.

[14] Khoury T, Chadha K, Javle M (2005). Expression of intestinal trefoil factor (TFF-3) in hepatocellular carcinoma. Int J Gastrointest Cancer.

[15] Shukla A, Gupta P, Singh R (2018). Glycolytic inhibitor 2-deoxy-d-glucose activates migration and invasion in glioblastoma cells through modulation of the miR-7-5p/TFF3 signaling pathway. Biochem Biophys Res Commun.

[16] Shang Yun-Li (2014). Clinical significance of expression of trefoil factor 3 in hepatocellular carcinoma. World Chinese Journal of Digestology.

[17] Lau WH, Pandey V, Kong X (2015). Trefoil factor-3 (TFF3) stimulates de novo angiogenesis in mammary carcinoma both directly and indirectly via IL-8/CXCR2. PLoS One.

[18] Guleng B, Han J, Yang JQ (2012). TFF3 mediated induction of VEGF via hypoxia in human gastric cancer SGC-7901 cells. Mol Biol Rep.

[19] Chan MW, Chan VY, Leung WK (2005). Anti-sense trefoil factor family-3 (intestinal trefoil factor) inhibits cell growth and induces chemosensitivity to adriamycin in human gastric cancer cells. Life Sci.

[20] You ML, Chen YJ, Chong QY (2017). Trefoil factor 3 mediation of oncogenicity and chemoresistance in hepatocellular carcinoma is AKT-BCL-2 dependent. Oncotarget.

[21] Zhao S, Han J, Zheng L (2015). Microrna-203 regulates growth and metastasis of breast cancer. Cell Physiol Biochem.

[22] Deng B, Wang B, Fang J (2016). MiRNA-203 suppresses cell proliferation, migration and invasion in colorectal cancer via targeting of EIF5A2. Sci Rep.

[23] Lohcharoenkal W, Harada M, Lovén J (2016). MicroRNA-203 inversely correlates with differentiation grade, targets c-MYC, and functions as a tumor suppressor in cSCC. J Investig Dermatol.

[24] Zhu X, Er K, Mao C (2013). miR-203 suppresses tumor growth and angiogenesis by targeting VEGFA in cervical cancer. Cell Physiol Biochem.

[25] Xu J, Zhu X, Wu L (2012). MicroRNA-122 suppresses cell proliferation and induces cell apoptosis in hepatocellular carcinoma by directly targeting Wnt/β-catenin pathway. Liver Int.

[26] Zhu J, Zheng Z, Wang J (2014). Different miRNA expression profiles between human breast cancer tumors and serum. Front Genet.

[27] Zhu YQ, Tan XD (2005). TFF3 modulates NF-κB and a novel negative regulatory molecule of NF-κB in intestinal epithelial cells via a mechanism distinct from TNF-α. Am J Phys Cell Phys.

[28] Loos M, De Creus A, Thim L, Remaut E, Rottiers P (2007). Murine trefoil factor 3 does not directly modulate LPS-mediated dendritic cell function. Scand J Immunol.

[29] Wei J, Huang X, Zhang Z (2013). MyD88 as a target of microRNA-203 in regulation of lipopolysaccharide or Bacille Calmette-Guerin induced inflammatory response of macrophage RAW264. 7 cells. Mol Immunol.

[30] Zhou M, Chen J, Zhou L (2014). Pancreatic cancer derived exosomes regulate the expression of TLR4 in dendritic cells via miR-203. Cell Immunol.

[31] Xu T, Chu Q, Cui J, Zhao X (2018). The inducible microRNA-203 in fish represses the inflammatory responses to gram-negative bacteria by targeting IL-1 receptor-associated kinase 4. J Biol Chem.

[32] Guo J, Sun M, Teng X (2017). MicroRNA-7-5p regulates the expression of TFF3 in inflammatory bowel disease. Mol Med Rep.

[33] Tarek M, Louka ML, Khairy E (2017). Role of microRNA-7 and selenoprotein P in hepatocellular carcinoma. Tumor Biol.

[34] Wu W, Liu S, Liang Y (2017). MiR-7 inhibits progression of hepatocarcinoma by targeting KLF-4 and promises a novel diagnostic biomarker. Cancer Cell Int.

